# Intravaginal Device-Type and Treatment-Length for Ovine Estrus Synchronization Modify Vaginal Mucus and Microbiota and Affect Fertility

**DOI:** 10.3390/ani8120226

**Published:** 2018-11-29

**Authors:** Paula Martinez-Ros, Marta Lozano, Fernando Hernandez, Alejandra Tirado, Alejandro Rios-Abellan, Maria Carmen López-Mendoza, Antonio Gonzalez-Bulnes

**Affiliations:** 1 Dpto. Produccion y Sanidad Animal, Facultad de Veterinaria, Universidad Cardenal Herrera-CEU, CEU Universities, C/Tirant lo Blanc, 7, 46115 Alfara del Patriarca, Valencia, Spain; marta.lozano1@uchceu.es (M.L.); rioabeale@alumnos.uchceu.es (A.R.-A.); clopez@uchceu.es (M.C.L.-M.); 2 Granja Cerromonte SL, 05358 San Juan de la Encinilla, Ávila, Spain; granja@cerromonte.es (F.H.); alejandra@cerromonte.es (A.T.); 3 Dpto. de Reproduccion Animal, INIA, Avda. Puerta de Hierro s/n., 28040 Madrid, Spain; bulnes@inia.es; 4 Dpto. de Toxicologia y Farmacologia, Facultad de Veterinaria, UCM, Ciudad Universitaria s/n., 28040 Madrid, Spain

**Keywords:** estrus synchronization, fertility, sheep, vaginal microbiota, vaginitis

## Abstract

**Simple Summary:**

This study examined the effects of different intravaginal device types used for estrous cycle management in sheep, and the timing of their insertion, on vaginal features (characteristics of vaginal mucus discharge, pH and microbiota) and fertility under field conditions.

**Abstract:**

Induction and synchronization of estrus and ovulation in sheep is based on intravaginal progestagen-impregnated polyurethane sponges or progesterone-loaded silicon-based devices (CIDR), in either short- (6–7 days) or long-term (12–14 days) protocols. Bearing in mind that the use of intravaginal sponges in long-term protocols has been related to the presence of vaginitis at removal, we compared the effects of sponges and CIDRs, maintained during either 7 or 14 days, on vaginal features (characteristics of vaginal mucus discharge, pH and microbiota) and fertility under field conditions. Almost all the ewes treated with intravaginal sponges showed vaginal discharge at device withdrawal, which was purulent and/or bloody in around 15% and 80% of the females treated for 7 and 14 days, respectively. The vaginal pH and microbiota changed in both groups when compared to control sheep, especially in ewes treated for 14 days, which showed a pH value around 8 and a higher incidence of *Salmonella* spp. and *Staphylococcus aureus*. On the other hand, independently of the length of the treatment, only around 15–20% of the sheep treated with CIDRs evidenced vaginal discharge (*p* < 0.00005 when compared to sponge groups), and such discharge was scarce, clear, and showed no changes in vaginal pH and microbiota when compared to control sheep. Fertility yields were associated with vaginal features, being higher in both short-term treatments (75%) and the long-term CIDR-based treatment (70%) than in the long-term sponge-based treatment (45%).

## 1. Introduction

Induction and synchronization of estrus and ovulation for reproductive management of sheep are traditionally based on the intravaginal insertion of progestagen-impregnated polyurethane sponges for 12–14 days, followed by the intramuscular injection of equine chorionic gonadotrophin (eCG) at sponge removal [1]. The use of intravaginal sponges for 12–14 days was early related to the presence of abnormal (purulent and/or hemorrhagic) and fetid vaginal discharges at their removal, which were associated with lower pregnancy rates [2]. The causes may be mainly related to a physical effect causing a constant retention of the vaginal secretions when intravaginal sponges are maintained for long periods of time [3], which may create a predisposition to proliferation and changes in the composition of the local microbiota [4,5]. These changes in the vaginal microbiota induce inflammation and infection associated with abnormal discharges [6]. Moreover, in addition to effects on fertility yields, the induction of vaginitis constitutes an issue opposite to the principles of animal welfare.

A possible alternative for minimizing the physical effects of the intravaginal sponge is the use of a different intravaginal device, named CIDR^®^ (Controlled Internal Drug Releasing device, Pfizer Inc., New York, NY, USA), which has been recently authorized for commercialization in the European Union. The CIDR is a Y-shaped silicon-based device and, due to its design and composition, which allows the drainage of any secretion, the rates of device loss, adherence and vaginitis are much lower than for sponges [7]. However, to the best of our knowledge, a direct comparison of vaginal features and microbiology among control non-treated sheep and sheep treated with sponges or CIDRs has not been performed yet.

In recent years, regardless of the intravaginal device used, the protocols for induction and synchronization of ovulations are evolving to shorter treatments, around 6–7 days of treatment with the intravaginal device [8,9,10]. These protocols have been related to a higher fertility after artificial insemination by laparoscopy [11]. A shorter period of treatment with the intravaginal device should be related to a lower incidence of vaginal inflammation and infection, presumptively leading to better fertility yields after natural breeding or cervical insemination. However, again, there are no comparative studies between different periods of treatment with the devices mentioned. 

Hence, bearing in mind the future availability of CIDR in the European Union and looking for a complete picture of the scenario, the objective of the present experiment was to compare vaginal features (characteristics of vaginal mucus discharge, pH and bacteriology) and fertility among non-treated control ewes and ewes treated with different devices (sponge or CIDR) and treatment lengths (14 or 7 days). Our hypothesis was that the type of intravaginal device and the duration of the treatment would affect physical characteristics and microbiota of vaginal mucus, also affecting fertility afterwards.

## 2. Materials and Methods

The study was performed during the breeding season (March) on a single commercial farm (Granja Cerromonte, San Juan de la Encinilla, Avila, Spain; latitude of 40.9° N), according to the Spanish Policy for Animal Protection RD53/2013, which complies with the European Union Directive 2010/63/UE about the protection of animals used for research. The study protocol was reviewed by the Committee of Ethics in Animal Research of the Instituto Nacional de Investigación y Tecnología Agraria y Alimentaria (INIA) and no ethical implications were observed.

### 2.1. Animals, Experimental Procedure, and Sampling

The experimental group consisted of 100 Lacaune multiparous sheep (2 to 5 years old), housed indoors with outdoor access. All the animals were monitored for adequate health status and specific pathogens, and clinical examinations were performed daily during the period of insertion of the devices by the farm’s veterinarian; no signs of disease were found in any of the animals. The experimental group remained together but, for experimental purposes, it was divided into five equal subgroups (*n* = 20 each one), based on the intravaginal device type (sponge, CIDR or none) and treatment length (14-day, 7-day or none) used for synchronization of ovarian activity, estrus and ovulation. Hence, 40 sheep were treated with one progestagen-impregnated sponge (Chronogest^®^, MSD Animal Health, Madrid, Spain) for either fourteen (group SP-14; *n* = 20) or seven days (group SP-7; *n* = 20), while 40 sheep were treated with one progesterone-loaded CIDR (CIDR^®^ Ovis, Zoetis, Madrid, Spain) for either fourteen (group CIDR-14; *n* = 20) or seven days (group CIDR-7; *n* = 20), and 20 ewes remained as untreated controls (group CON-0). At intravaginal device removal, groups SP and CIDR received one intramuscular injection of 400 IU of eCG (Foligon^®^, MSD Animal Health, Madrid, Spain), while short-term treatments (groups SP-7 and CIDR-7) also received an intramuscular injection of 5 mg of prostaglandin F_2α_ (Dinolytic^®^, Zoetis, Madrid, Spain). 

**Figure 1:**
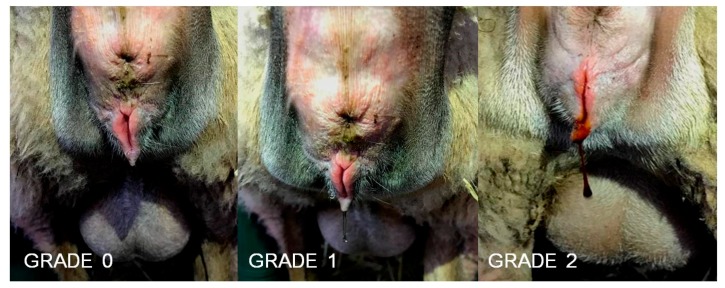
Scoring of vaginal discharge at intravaginal device removal: 0—negligible or no discharge; 1—some amount but clear discharge; 2—abundant amounts and hemorrhagic or purulent.

The intravaginal device was withdrawn on the same day in all the animals and therefore, animals in short treatments had 7 days of treatment with the device, animals in long treatments had 14 days, while control animals had zero days. At intravaginal device withdrawal, characteristics of vaginal discharge (i.e., amount, odor, whether purulent or hemorrhagic) were scored (0—negligible or no discharge; 1—some amount but clear discharge; 2—abundant amounts and hemorrhagic or purulent; Figure 1). Immediately, a sterile swab (Cary Blair sterile transport swabs, Deltalab, Barcelona, Spain) was used to collect mucus samples from the posterior region of the vagina (4 to 5 cm into the vagina), which were sent to the laboratory for bacterial culture. Finally, vaginal mucus pH was evaluated using pH-indicator strips (Merck KGaA, 64,271 Damstadt, Germany; working range pH 6.5–10.0).

After sampling the ewes, males were introduced in a ratio of 1:5 and allowed to remain with the females for 28 days to include possible return estrus. Fertility rate was assessed by transabdominal ultrasonography (NanoMaxx, Sonosite, Bothell, WA, USA), performed at Days 38 and 67 after device withdrawal for assessing induced and return estrus, and confirmed at lambing.

### 2.2. Bacteriology

Swabs were used to plate samples on different selective media (all from Scharlab, Barcelona, Spain), in order to determine the presence or absence of several bacteria. The presence of *Staphylococcus aureus* was determined by using Baird Parker agar and incubation at 37 °C for 24 h. Typical colonies were confirmed by coagulase test [12]. The presence of *Escherichia coli* and *Klebsiella* spp. was determined using violet red bile glucose agar plates, which were incubated at 37 °C for 48 h [13]. Typical colonies were afterwards transferred to Levine agar and incubated at 45 °C for 24 h for *E. coli* isolation, and to MacConkey agar for *Klebsiella* spp. isolation, incubating at 37 °C for 48 h [12]. MacConkey agar was also used for isolation of *Aeromonas* spp., incubating at 37 °C for 48 h [14], while *Shigella* spp. and *Salmonella* spp. were cultured on xylose lysine deoxicholate agar (XLD agar) and incubated at 37 °C for 24 h [12]. In all cases, colonies were identified by morphological characteristics.

### 2.3. Statistical Analysis

Statistical analysis was performed using SPSS^®^ 22.0 (IBM Corporation, New York, NY, USA). The effects of the intravaginal device type (sponge, CIDR or none) and the treatment length (14-day, 7-day or none) on vaginal features at device removal (characteristics of vaginal mucus discharge, pH and bacteriology) and subsequent fertility were assessed by analyses of variance (ANOVA). Statistical analysis of results expressed as percentages was performed after arcsine transformation of the values for each individual percentage. All results are expressed as mean ± S.E.M. and the statistical significance was accepted at *p* < 0.05, with *p* < 0.1 being considered a trend.

## 3. Results

At removal of the intravaginal device, almost all ewes in the groups SP-7 and SP-14 (100% and 94.4%, respectively) showed vaginal discharge (Table 1); a percentage significantly higher than in the groups CIDR-7 and CIDR-14 (20.0% and 15.0%, respectively; *p* < 0.00005 for the comparisons among SP and CIDR groups). The discharge was scarce, clear and mucous in females treated with CIDR; conversely, 10.5 and 83.3% of the females in the groups SP-7 and SP-14, respectively, showed purulent and/or bloody vaginal discharges (*p* < 0.00005 when comparing SP-7 and SP-14 and *p* = 0.06 and *p* < 0.00005 when comparing the CIDR groups with SP-7 and SP-14, respectively).

Assessment of vaginal pH at device removal also showed significant differences among treatments. The values were similar, around 6.8–6.9, in the controls CON-0 and the CIDR groups (*p* > 0.818). Conversely, the value for pH increased in both SP groups, with significant differences when assessing the effect of duration of the treatment. Hence, SP-7, with a pH of 7.2 ± 0.1, had a trend for higher values than CON-0 (*p* = 0.07), CIDR-7 (*p* < 0.05) and CIDR-14 (*p* = 0.05), while the differences were highly significant when assessing the SP-14 group, with a pH of 8.0 ± 0.2, against SP-7 (*p* < 0.001) and CON-0, CIDR-7 and CIDR-14 (*p* < 0.00001 for all).

The prevailing bacteria (Table 2) in the CON-0 group were the Gram negative (G–) *E. coli*, *Aeromonas* spp. and *Shigella* spp. (84.2, 84.2 and 68.4% of the ewes, respectively), and the Gram positive (G+) *S*. *aureus* (68.4% of the ewes). Assessment of the animals with intravaginal treatments showed similar prevalence for *E. coli*. However, all the treated groups showed appearance of the G– *Salmonella* spp. (absent in CON-0, but 5% in CIDR groups and around 11.1% in SP groups), although differences only showed a trend for SP groups when compared to CON-0 (*p* = 0.07). The SP-14 group also showed an increase in the appearance of *S. aureus, Shigella* spp. and the G– *Klebsiella* spp. (84.2, 73.7 and 10.5% of the ewes, respectively); however, there was only statistical significance in the case of *S. aureus* (*p* < 0.05). Finally, the CIDR groups showed a significantly decreased presence of *Aeromonas* spp. (65.0% for both CIDR-7 and CIDR-14; *p* < 0.05 when compared to CON-0 and SP groups).

Assessment of fertility at induced estrus also showed significant differences among treatments (Table 3). Pregnancy in response to the hormonal treatment was overall higher in both short-term treatments (75% for the groups CIDR-7 and SP-7) and the long-term CIDR-based treatment (70% for the group CIDR-14) than in the long-term sponge-based treatment (45% for the group SP-14; *p* < 0.05 when compared with all the other groups). Moreover, it was detected that 15% of the sheep in the group SP-14 resulted pregnant after displaying a short estrous cycle in response to the treatment; a finding which was not identified in the other groups. As shown in Table 3, fertility to both synchronized and returning estruses was similar among the different groups (90% for CIDR-7, 80% for SP-7, 90% for CIDR-14 and 75% for SP-14). There were significantly lower percentages of sheep in the control group which became pregnant around the induced and returning estrus of the treated ewes (15 and 60%, respectively; *p* < 0.05 when compared with all the other groups).

## 4. Discussion

The results of the present study indicate that both the type of intravaginal device (sponge or CIDR) and the duration of the progestative treatment (14 or 7 days) may affect characteristics of vaginal mucus discharge, pH and bacteriology, and fertility.

Almost all the ewes treated with intravaginal sponges showed vaginal discharge at device withdrawal. The characteristics of such vaginal discharge were different depending on the duration of the sponge insertion. Around 80% of the sheep treated with classical long-term treatments (14-day) showed purulent and/or bloody vaginal discharges. These results reinforce previous studies reporting that long-term progestogen treatments are associated with soaking of the polyurethane sponge, retention of vaginal secretions and abnormal discharges at sponge withdrawal [3]. Hemorrhagic and purulent discharges are associated with histological and cytological alterations in the vaginal wall of treated ewes (epithelial hyperplasia and hypertrophy, hemorrhage and perivascular infiltrate and increased number of epithelial cells, neutrophils, macrophages and erythrocytes [15]). On the other hand, the percentage of ewes showing purulent and/or bloody vaginal discharges decreased to around 10% when applying short-term protocols (7-day), which highlights the importance of the duration of sponge insertion. Our current results, although without evaluating innate immune variables (e.g., pro-inflammatory cytokines and acute phase proteins), therefore give evidence that the use of short-term protocols may alleviate the problems previously described in the case of long-term protocols, improving health and welfare conditions in animals treated with intravaginal sponges.

The assessment of vaginal discharges at device removal showed that only around 15–20% of the sheep treated with CIDRs had a discharge. In all the cases, such discharges were scarce, clear and mucous. Hence, in agreement with previous studies [7], the design and composition of the CIDR allows the drainage of any secretion, avoiding retention and subsequent changes in vaginal fluids. The duration of insertion of the CIDR did not affect the characteristics of the vaginal discharge, conversely to polyurethane sponges, presumably because of non-blockage of vaginal fluids.

Previous studies have also shown that abnormal vaginal discharges after long-term progestogen treatments are the result of inflammatory and infectious processes caused by changes in the vaginal microbiota [4,6]. Although non-quantitative, the results of the current trial indicated that, compared to control ewes, in which vaginal microbiota was similar to described in previous studies [16], the sheep treated with sponge for 14 days showed a higher incidence of *Salmonella* spp. and especially *Staphylococcus aureus*. Both *Salmonella* spp. and *S. aureus* have been previously related to aerobic vaginitis causing increased inflammatory immune response [17,18] and, specifically for sheep, subsequent reproductive problems [19,20]. Particularly, *S. aureus* is considered an inhabitant of vulva and vagina and identified as the most common causal agent of purulent vaginitis in ewes [18,21], which is consistent with the presence of purulent vaginal discharges at sponge removal. Although it was not the case in the present trial, there are previous studies reporting that sheep treated with either sponges or CIDR have a high prevalence of *E. coli*, probably of fecal source, which are opportunistic agents of bacterial vaginitis [5].

On the other hand, in the present study, we have to highlight that the groups treated with CIDRs, either for 7 or 14 days, showed a significantly decreased presence of *Aeromonas* spp. This is a surprising finding and we have actually not found a possible explanation for this change in the vaginal microbiota of sheep treated with CIDRs. A possible hypothesis may be based in the facts that *Aeromonas* spp. are ubiquitous in water and usually are associated with infections by exposure to contaminated water, while exposition to progesterone may decrease the water content of vaginal mucus by approximately 90% [22].

A possible option to avoid vaginitis, and further changes in vaginal features and fertility, in ewes treated with polyurethane sponges would be the use of an adequate prophylactic management that can include the disinfection of the vulvar area and all the material used for the application of the devices, and even the use of local and/or systemic antibiotics [5,23]. However, this is opposite to the current principle of limitations in the use of antimicrobials and is unnecessary if using short-term sponge-based or CIDR-based protocols.

The changes in the vaginal secretion and microbiota found in sponge-treated sheep may be related to changes in the vaginal pH at device removal. Controls and ewes treated with CIDRs, either with short- or long-term protocols, showed near-neutral pH similarly to that previously described for non-treated sheep [16]. The neutral pH protects the vagina from pathogenic microorganisms, which grow best at pH higher than 7.5 [24,25]. Conversely, sheep treated with short-term and long-term sponge-based protocols showed a significant increase in the vaginal pH. Such increases were higher in ewes exposed to long-term sponge-based treatments, reaching an average of 8.0; in the case of females showing hemorrhagic discharges at sponge removal, this finding may be related to alkalinity of the blood. In any case, increases in the vaginal pH increase susceptibility to infections, which can also indirectly affect fertility [22]. 

Our results for fertility are consistent with data for vaginal discharge characteristics. Fertility yields were similar among sheep treated with CIDR, in both short- and long-term protocols, and sheep treated with sponges in short-term protocols, in agreement with previous studies [26,27,28]. These yields were similar to what was expected in these groups; however, sheep treated with sponges in long-term protocols had lower yields. The difference was not statistically significant, which may be a statistical artifact due to the number of ewes used, but it is real and of practical impact. We cannot elucidate the causes for this lower fertility under the condition of the present study but, bearing in mind the inexistence of reproductive problems in the flock and the fact that males were the same for all the groups, we can hypothesize an adverse effect on sperm viability from the inflammatory vaginal environment, or the existence of failures in the ovarian response to the long-term sponge treatment. Previous studies have reported abnormal follicular development with large persistent follicles [29,30]; a hypothesis which may be supported by the existence of at least 15% of sheep displaying a short estrous cycle in this group.

## 5. Conclusions

Almost all the ewes treated with intravaginal sponges showed vaginal discharge at device withdrawal, which was purulent and/or bloody in around 80% of the females treated for 14 days and around 15% of the females treated for 7 days. The vaginal pH and microbiota changed in both groups when compared to control ewes, especially in the long-term protocol, where pH was around 8 and the ewes showed a higher incidence of *S. aureus* and a decrease in fertility. On the other hand, only around 15–20% of the sheep treated with CIDRs, independently of the length of the treatment, evidenced vaginal discharge (which was scarce, clear and mucous in all the cases), and both CIDR groups showed a vaginal pH and microbiota similar to control sheep.

## Figures and Tables

**Table 1:** Characteristics of vaginal mucus discharge (0—negligible or no discharge; 1—some amount but clear discharge; 2—abundant amounts and hemorrhagic or purulent) and pH at intravaginal device withdrawal in sheep treated with one progestagen-impregnated sponge for either 14 (group SP-14; *n* = 20) or 7 days (group SP-7; *n* = 20), one progesterone-loaded CIDR for either 14 (group CIDR-14; *n* = 20) or 7 days (group CIDR-7; *n* = 20), or no treatment (group CON-0; *n* = 20).

Characteristics	SP-7	SP-14	CIDR-7	CIDR-14	CON-0
Vaginal mucus discharge (grade 0, %)	0	5.6	80	85	100
Vaginal mucus discharge (grade 1, %)	89.5	11.1	20	15	0
Vaginal mucus discharge (grade 2, %)	10.5	83.3	0	0	0
pH	7.2 ± 0.1	8.0 ± 0.2	6.8 ± 0.1	6.9 ± 0.2	6.9 ± 0.2

**Table 2:** Prevailing vaginal flora (%) at intravaginal device withdrawal in sheep treated with one progestagen-impregnated sponge for either 14 (group SP-14; *n* = 20) or 7 days (group SP-7; *n* = 20), one progesterone-loaded CIDR for either 14 (group CIDR-14; *n* = 20) or 7 days (group CIDR-7; *n* = 20), or no treatment (group CON-0; *n* = 20).

Bacteria	SP-7	SP-14	CIDR-7	CIDR-14	CON-0
*Aeromonas* spp. (%)	88.9	89.5	65.0	65.0	84.2
*Escherichia coli* (%)	90.0	85.0	84.2	83.3	84.2
*Klebsiella* spp. (%)	5.6	10.5	5.0	5.0	5.3
*Salmonella* spp. (%)	11.1	10.5	5.0	5.0	0.0
*Shigella* spp. (%)	66.7	73.7	60.0	65.0	68.4
*Staphylococcus aureus* (%)	61.1	84.2	65.0	65.0	68.4

**Table 3:** Fertility obtained around synchronized and return estrus in sheep treated with one progestagen-impregnated sponge for either 14 (group SP-14; *n* = 20) or 7 days (group SP-7; *n* = 20), one progesterone-loaded CIDR for either 14 (group CIDR-14; *n* = 20) or 7 days (group CIDR-7; *n* = 20), or no treatment (group CON-0; *n* = 20).

Fertility	SP-7	SP-14	CIDR-7	CIDR-14	CON-0
Number of ewes (*n*)	20	20	20	20	20
Ewes pregnant at induced estrus (*n* and %)	15 (75%)	9 (45%)	15 (75%)	14 (70%)	3 (15%)
Ewes pregnant at return estrus (*n* and %)	1 (5%)	6 (30%)	3 (15%)	4 (20%)	9 (45%)
Ewes non-pregnant (*n* and %)	4 (20%)	5 (25%)	2 (10%)	2 (10%)	8 (40%)
Ewes lambing (*n* and %)	16 (80%)	15 (75%)	18 (90%)	18 (90%)	12 (60%)

## References

[1] Abecia J.A., Forcada F., Gonzalez-Bulnes A. (2012). Hormonal control of reproduction in small ruminants. Anim. Reprod. Sci..

[2] Scudamore C.L. (1988). Intravaginal sponge insertion technique. Vet. Rec..

[3] Al-Hamedawi T.M., Khammas D.J., Al-Ubaidi A.S. (2003). Effect of estrus synchronization on vaginal flora and subsequent fertility in ewes. Iraqi J. Vet. Sci..

[4] Sargison N.D., Howie F., Mearns R., Penny C.D., Foster G. (2007). Shiga toxin-producing *Escherichia coli* as a perennial cause of abortion in a closed flock of Suffolk ewes. Vet. Rec..

[5] Vasconcelos C., Brandão F.Z., Martins G., Penna B., Souza J.M.G., Lilenbaum W. (2016). Qualitative and quantitative analysis of bacteria from vaginitis associated with intravaginal implants in ewes following estrus synchronization. Ciência Rural.

[6] Martins G., Figueira B., Penna B., Brandão F., Varges R., Vasconcelos C., Lilenbaum W. (2009). Prevalence and antimicrobial susceptibility of vaginal bacteria from ewes treated with progestin-impregnated intravaginal sponges. Small Rumin. Res..

[7] Suarez G., Zunino P., Carol H., Ungerfeld R. (2006). Changes in the aerobic vaginal bacterial mucous load and assessment of the susceptibility to antibiotics after treatment with intravaginal sponges in anestrous ewes. Small Rumin. Res..

[8] Menchaca A., Rubianes E. (2004). New treatments associated with timed artificial insemination in small ruminants. Reprod. Fertil. Developm..

[9] Letelier C.A., Contreras-Solis I., García-Fernández R.A., Ariznavarreta C., Tresguerres J.A., Flores J.M., Gonzalez-Bulnes A. (2009). Ovarian follicular dynamics and plasma steroid concentrations are not significantly different in ewes given intravaginal sponges containing either 20 or 40 mg of fluorogestone acetate. Theriogenology.

[10] Cox J.F., Allende R., Lara E., Leiva A., Díaz T., Dorado J., Saravia F. (2012). Follicular dynamics, interval to ovulation and fertility after AI in short-term progesterone and PGF2α oestrous synchronization protocol in sheep. Reprod. Dom. Anim..

[11] Dos Santos-Neto P.C., García-Pintos C., Pinczak A., Menchaca A. (2015). Fertility obtained with different progestogen intravaginal devices using short-term protocol for fixed-time artificial insemination (FTAI) in sheep. Livestock Sci..

[12] Murray P.R., Baron E.J., Jorgensen J.H., Pfaller M.A., Yolken R.H. (2003). Manual of clinical microbiology.

[13] Mossel D.A.A. (1985). Media for Enterobacteriaceae. Int. J. Food Microbiol..

[14] Desmond E., Janda J.M. (1986). Growth of Aeromonas species on enteric agars. J. Clin. Microbiol..

[15] Manes J., Campero C., Hozbor F., Alberio R., Ungerfeld R. (2015). Vaginal histological changes after using intravaginal sponges for oestrous synchronization in anoestrous ewes. Reprod. Domest. Anim..

[16] Swartz J.D., Lachman M., Westveer K., O’Neill T., Geary T., Kott R.W., Berardinelli J.G., Hatfield P.G., Thomson J.M., Roberts A. (2014). Characterization of the vaginal microbiota of ewes and cows reveals a unique microbiota with low levels of lactobacilli and near-neutral pH. Front. Vet. Sci..

[17] Ley W.B., Bowen J.M., Mathewson J.J. (1980). Salmonella-induced vaginitis. Vet. Med..

[18] Donders G.G.G., Vereecken A., Bosmans E., Dekeersmaecker A., Salembier G., Spitz B. (2002). Definition of a type of abnormal vaginal flora that is distinct from bacterial vaginosis: Aerobic vaginitis. Br. J. Obstetr. Gynecol..

[19] Shallali A.A., Hussein A.M., Salih M.M., Dafalla E.A. (2001). A preliminary report on bacteria isolated from the female genital tract of Sudanese sheep and goats. Sudan J. Vet. Res..

[20] Cortés-López N.G., Abad-Zavaleta J., Bravo-Delgado H.R., Meza-Villalvazo V.M., Sachman-Ruiz B., García-Arellano C., Ventura S.T. (2013). Effect of fluorogestone acetate on the vaginal microbiota from ewes Pelibuey in the Papaloapan region. Trop. Subtrop. Agroecosyst..

[21] Bragança J.F.M., Maciel J.M., Girardini L.K., Machado S.A., da Rocha J.F.X., Tonin A.A., da Rocha R.X. (2017). Influence of a device intravaginal to synchronization/induction of estrus and its reuse in sheep vaginal flora. Comp. Clin. Pathol..

[22] Nakano F.Y., de Barros R., Leão F., Sandro C., Esteves S.C. (2015). Insights into the role of cervical mucus and vaginal pH in unexplained infertility. Med. Express.

[23] Gatti M., Zunino P., Ungerfeld R. (2011). Changes in the aerobic vaginal bacterial mucous load after treatment with intravaginal sponges in anoestrous ewes: Effect of medroxiprogesterone acetate and antibiotic treatment use. Reprod. Dom. Anim..

[24] Cohen L. (1969). Influence of pH on vaginal discharges. Br. J. Vener. Dis..

[25] Roy S., Caillouete J.C., Roy T., Faden J.S. (2004). Vaginal pH is similar to follicle stimulating hormone for menopause diagnosis. Am. J. Obstetr. Gynecol..

[26] Ungerfeld R., Rubianes E. (2002). Short term priming with different progestogen intravaginal devices (MAP, FGA and CIDR) for eCG-estrous induction in anestrous ewes. Small Rum. Res..

[27] Ozyurtlu N., Kucukaslan I., Cetin Y. (2010). Characterization of oestrous induction response, oestrous duration, fecundity and fertility in Awassi ewes during the non-breeding season utilizing both CIDR and intravaginal sponge treatments. Reprod. Dom. Anim..

[28] Swelum A.A., Alowaimer A.N., Abouheif M.A. (2015). Use of fluorogestone acetate sponges or controlled internal drug release for estrus synchronization in ewes: Effects of hormonal profiles and reproductive performance. Theriogenology.

[29] Johnson S.K., Dailey R.A., Inskeep E.K., Lewis P.E. (1996). Effect of peripheral concentrations of progesterone on follicular growth and fertility in ewes. Dom. Anim. Endocrinol..

[30] Viñoles C., Meikle A., Forsberg M., Rubianes E. (1999). The effect of subluteal levels of exogenous progesterone on follicular dynamics and endocrine patterns during the early luteal phase of the ewe. Theriogenology.

